# Morphogenesis of Urban Water Distribution Networks: A Spatiotemporal Planning Approach for Cost-Efficient and Reliable Supply

**DOI:** 10.3390/e20090708

**Published:** 2018-09-14

**Authors:** Jonatan Zischg, Wolfgang Rauch, Robert Sitzenfrei

**Affiliations:** Unit of Environmental Engineering, University of Innsbruck, Technikerstraße 13, A-6020 Innsbruck, Austria

**Keywords:** urban transition, EPANET 2, network disconnection, network growth, uncertainties, performance and adaptation, flow entropy

## Abstract

Cities and their infrastructure networks are always in motion and permanently changing in structure and function. This paper presents a methodology for automatically creating future water distribution networks (WDNs) that are stressed step-by-step by disconnection and connection of WDN parts. The associated effects of demand shifting and flow rearrangements are simulated and assessed with hydraulic performances. With the methodology, it is possible to test various planning and adaptation options of the future WDN, where the unknown (future) network is approximated via the co-located and known (future) road network, and hence different topological characteristics (branched vs. strongly looped layout) can be investigated. The reliability of the planning options is evaluated with the flow entropy, a measure based on Shannon’s informational entropy. Uncertainties regarding future water consumption and water loss management are included in a scenario analysis. To avoid insufficient water supply to customers during the transition process from an initial to a final WDN state, an adaptation concept is proposed where critical WDN components are replaced over time. Finally, the method is applied to the drastic urban transition of Kiruna, Sweden. Results show that without adaptation measures severe performance drops will occur after the WDN state 2023, mainly caused by the disconnection of WDN parts. However, with low adaptation efforts that consider 2–3% pipe replacement, sufficient pressure performances are achieved. Furthermore, by using an entropy-cost comparison, the best planning options are determined.

## 1. Introduction

Cities are highly dynamic structures that are experiencing demographic changes with large population growth in most regions of Asia and Africa, whereas a general population decline is taking place in Europe. The shrinking city phenomenon and abandonment of urban districts are gaining in importance worldwide. According to Oswalt and Rieniets [1] every fourth city in the world was shrinking between 1990 and 2000 with a rising trend. One of the most prominent examples is the city of Detroit, USA, where today about one third of the town area is abandoned or demolished due to deindustrialization [2]. Conversely, strong city growth and the expansion of neighborhoods with annual growth rates up to 5% are observed in the last years [3]. 

These urban transitions stress not only the above-ground infrastructure like streets or public transport, but also the underground water infrastructure due to the necessary modifications in the network’s structure and function. Water supply systems are one of the most important infrastructures in society and its well-functioning is essential. Shifting demand patterns, endeavors from centralized to decentralized techniques [4], network expansions, or connectivity loss are only a few examples for transition processes which stress the ability to perform. Along with the long life span of several decades (in some cases even exceeding 100 years), the influence of a newly constructed pipe in the future system and the interaction with the existing system emphasize the importance of future oriented planning and management [5].

“Network transitioning” describes the pathway from an existing (source) network to a future (destination) network [6]. Usually transition processes of water infrastructure are relatively slow and take place over decades, where the network structure remains nearly constant (see e.g., Ref. [4]). Here we present a method and apply it to a case study to assess fast ongoing transitions, with severe impacts on the network’s structure. Changes of the network structure are densifications, expansions and/or disconnection of network parts caused by climate change, population changes, changes in land use, and shifts in water technology [7,8,9]. While new network parts must be designed for expected load conditions and spatial constraints, the existing network may need to be adapted to provide sufficient performances at all times of the transition process. In many cases, the detailed future network layout is largely unknown, because decisions on urban transitions are usually made without taking the water infrastructure into account. In summary, transitioning of urban infrastructure systems is characterized by real world complexity and high uncertainties, which is strongly dependent on the urban development.

In the field of water distribution system analysis, it is widely accepted that measures for hydraulic capacity reliability and redundancy have to be considered in the design process to account for such uncertainties and unforeseeable events. Among many stochastic and reliability-based methods, flow entropy, based on Shannon’s informational entropy [10], is proven to be a consistent surrogate measure of reliability and failure tolerance [11,12]. High entropy values indicate equally important flow paths, i.e., the absence of dominant supply structures, whose failure could cause a severe system breakdown. Higher entropies are not only dependent on the number of supply sources and alternative flow paths, but also on their scale and spatial location. For example, a loop in the network structure with high flows (e.g., proximity to the supply source) is rated higher than a loop in secondary pipes with lower flows, where potential system failures have smaller impacts on the global performance. Compared to other criteria, this measure has the advantage that it is relatively easy to calculate, non-iterative, and that the data requirements are minimal [11,13]. 

Previous studies presented methods of designing and evaluating the long-term transition under deep uncertainties of water infrastructure (planning horizon of several decades), as for example shown by Marlow et al. [14], Eggimann et al. [15] and Urich and Rauch [16] whose work emphasized on urban drainage system transitions. Creaco et al. in Refs. [17,18] presented an optimization methodology for the optimal growth of water distribution networks (WDNs) with respect to costs and reliability, also considering demand uncertainties. In their work, they considered the step-by-step connection of pipes and concluded that solely designing one ideal “final state network” is not optimal, neglecting possible deficiencies of the WDN at intermediate states. However, disconnections and uncertainties of the network topology were not considered in their works. Existing studies of complex real world WDN transitions are limited and therefore further research is necessary also with regard to exploring case studies of unspecific methods [19,20]. A promising concept is the creation of many possible future network layouts, rather than assessing a fixed topology. The idea is to use the often available future road network (RN) from city planners as the basis for an automatic WDN planning approach. This concept of so-called “in-silico” or “semi-virtual” networks was already used for network reconstructions [21] or even the approximation of real water infrastructure networks where missing network information is approximated with known surrogate information, such as the co-located road network data [22,23]. 

In comparison with the work presented in Ref. [22], this study extends transition modeling by the interaction of the existing WDN data and future “in-silico” network data, also taking into account uncertain population developments that allow for future design under a probable range of uncertainties. Furthermore, the work presents a simple local adaptation concept of pipe replacement based on the least- “monetary” cost path which is applied to the WDN, in case a defined critical performance can no longer be achieved at a certain transition state. After sufficient performances are achieved for all WDN states, we perform a cost-reliability comparison of different WDN design alternatives (layout and design demand). Entropy-cost ratios help decision makers identify reliable planning options that work for a broad range of future conditions, and quantify necessary costs (WDN layout and adaptation costs) at an early state of the transition. 

The novelty of this work is to investigate many planning options with regard to WDN structure, and to provide sufficiently working, reliable and cost-efficient systems over time. Specific problem states can be well-defined and optimized, but in reality, transition processes are often driven by a multitude of uncertain conditions. Therefore, the proposed approach serves as a screening method for decision makers to realize a masterplan and gives a holistic view on network dynamics, rather than focusing on one specific problem or state. 

## 2. Materials and Methods

### 2.1. Model Development

**Figure 1:**
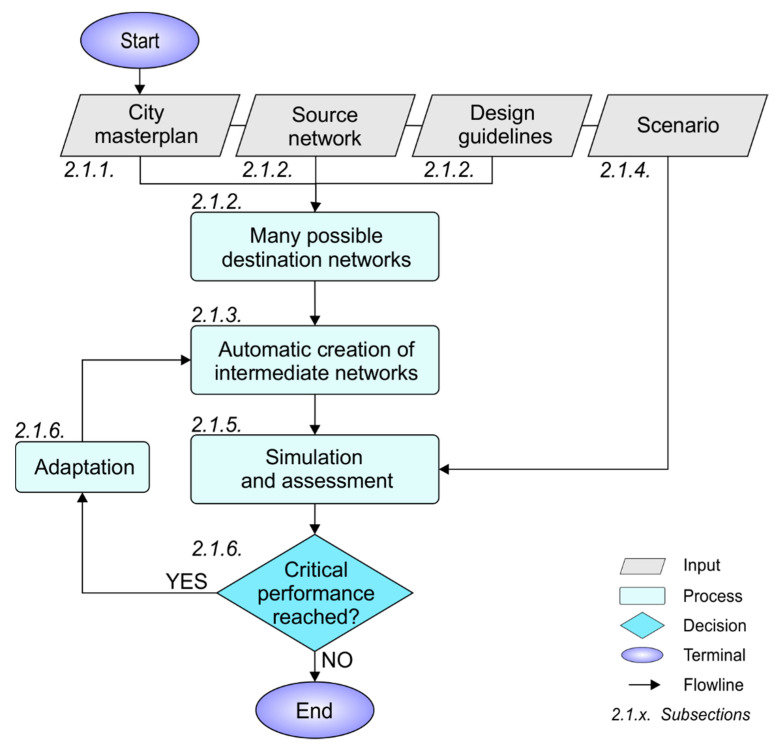
Description of the model for water distribution network (WDN) transitioning.

This section presents the development of the model to assess WDN transitioning, on the basis of the city masterplan, future network design alternatives and concepts for system adaptation. Additionally, all planning options are assessed by a variety of scenarios (e.g., demand increase due to population growth) during the transition process. In Figure 1 the model is shown in a flow chart and described in detail in the subsequent sections (see headed associated section numbers). 

#### 2.1.1. City Masterplan

In urban planning, the city masterplan comprises a description of the future city’s spatial and temporal development, defines regulatory changes, and lays out a strategic direction of future visions. Comprehensive objectives regarding urban landscape and water resource development, national and public interests are specified from a municipality perspective and therefore a masterplan is case study specific [24]. Such a blueprint for the future is the basis for the successful realization of a transition process, out of which various planning alternatives can be derived for further testing and optimization. Furthermore, it decreases the number of uncertainties and makes the modeling process less complex [25,26]. The masterplan defines, for example, ambitions from centralized to decentralized water management in the future, the disconnection of abandoned city districts due to water quality problems, expected city densifications or expanding neighborhoods, which lead to network growth. In the cases where no masterplan is available, stochastic network growth models such as random or fitness-based attachment models can be used to anticipate network development [27]. 

#### 2.1.2. Source and Destination Networks

**Figure 2:**
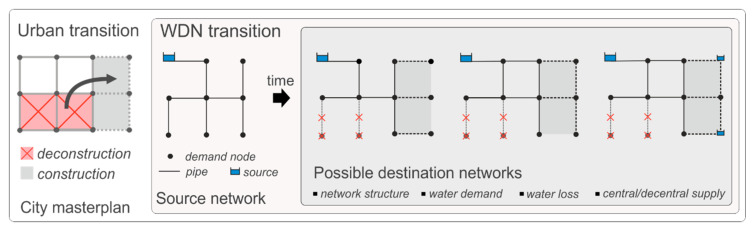
Conceptual representation of an urban transition affecting the water distribution: Source network and possible future destination networks.

The starting point of this modeling approach is an existing hydraulic model (e.g., EPANET 2 model) of the WDN (hereinafter called source network). It includes all water sources, pipes and junctions with their attributes, control devices and all information on spatially and time dependent water consumption and water leakage. While the source network represents an existing WDN, the future system may be fairly unknown in terms of a detailed network layout and future water demand (e.g., due to city expansions). Based on a city masterplan, a new future supply concept can be developed, considering, e.g., various future network topologies. In this process, many future systems (destination networks) with different graph theoretical and hydraulic design are created (e.g., loop degree of the network, design demand and supply redundancy). To account for future demand uncertainty in the design process, different pipe capacity factors (hereinafter called “safety factors”) are used. Compared to the source network, the destination network differs in its topology, demand distribution, pipe diameter & roughness, position of water sources and water losses (see Figure 2).

**Figure 3:**
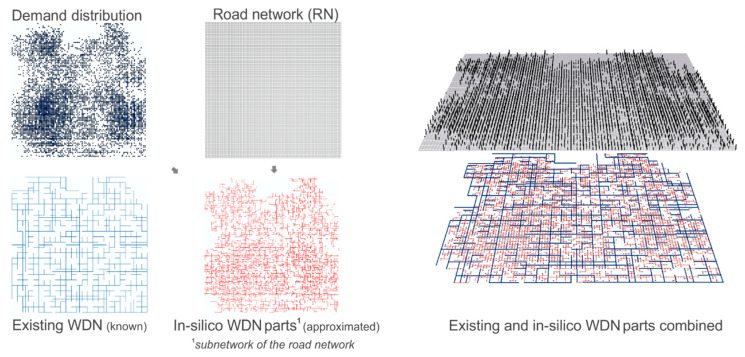
Example of an existing WDN layout and the approximation of unknown WDN parts using spatial road network data as surrogate.

Given a defined goal in the city masterplan, a set of destination networks is created. Thereby parts of the source network and the newly designed “in-silico” WDN parts are combined. The idea is to use the often available future road network (RN) from city planners as the basis for an automatic WDN planning approach. Previous studies proved the high co-location between road and urban infrastructure networks, where about 80% of WDNs are located below 50% of the RN [28,29]. In the presented model, it is assumed that 100% of the WDN is co-located with the RN (WDN ⊆ RN). However, not all roads may contain pipes; this is mainly due to cost reasons. So there are the two extreme cases of possible “in-silico” WDN topologies; (1) a branched network, representing the minimum spanning tree of the RN, and (2) a highly looped topology, where the WDN layout is identical with the RN. Between these two cases, the loop degree can vary with the cycle index (CI). While a CI of 0 describes a branched layout, a CI > 0 indicates the availability of alternative flow paths (loops) in the WDN, which is also an indicator for reliability and redundancy. More detailed information of the CI can be found in Mair et al. [29]. During the generation process of the future WDN parts, different CIs are considered. Furthermore, the digital elevation model (DEM), the existing WDN, (future) demand distribution, the position of (future) water sources and the (future) road network are required for model input (see Figure 3). The “in-silico” WDN parts can either represent missing data of existing systems, or possible future network expansions/densifications.

The WDN creation is performed with DynaMind, an open source software written in C++ and developed at the Unit for Environmental Engineering of the University of Innsbruck. The software contains (1) a spanning tree based algorithm for designing a minimal operating new network layout, (2) an algorithm to create a cyclic structure for the purpose of network reliability and (3) an automated pipe-sizing algorithm to create WDN based on geographic information system (GIS) data as input [30]. To obtain a connected network, the “in-silico” parts are connected to the closest junction of the existing WDN, unless other connection points are manually defined. The software uses a pipe sizing algorithm for selected pipe sets (e.g., all unknown WDN parts) based on a defined pressure surface inclination at hourly peak demand (*Q_h_,_max_*) as presented by Saldarriaga et al. [31]. Other WDN design optimization algorithms might be more suitable when focusing on the detailed planning of small case studies. However, the reasons for choosing this design algorithm are the low computational costs and the good approximation of optimal WDN design solutions, even for WDNs with several thousands of nodes and pipes.

#### 2.1.3. Intermediate State Networks

**Figure 4:**
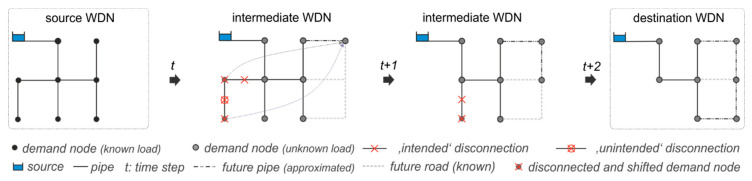
Automatic creation of intermediate WDN on basis of the source and destination WDN and the city masterplan.

In most cases, changes from an existing system to the final system are long term processes which cannot be directly implemented at one specific time step, but have to be successively introduced in different phases, making the modeling of intermediate transition states unavoidable [18]. During such a transition process the network’s structure and function changes progressively. Parts of the existing system may be taken out of service, while other parts are connected to the existing system or the demand pattern changes, e.g., due to changing land use. At the same time, the total water consumption and its spatial distribution can change over time. Such stresses can cause a system breakdown, where required performance levels cannot be guaranteed anymore. In our analysis several transition state models, which represent specific time steps during the investigation period, are automatically created (see Figure 4). 

The basis for the intermediate state model configurations is the source and the destination networks and the masterplan, which describes the spatiotemporal development of the city, and as well as the WDN. By disconnecting spatially defined pipes from the WDN (“intended disconnection”), the network may be split into sub-networks which are not connected to the water source anymore. This is the case when a pipe coincides with a bride or cut-edge of the network, whose deletion increases the number of connected components [32]. Resulting “unintended” disconnections are determined through a network connectivity analysis. Unconnected parts are either additionally removed, or reconnected to the main network, depending on the unintended and intended pipe disconnection ratio. Newly connected pipes have the attributes (location, connection points, diameter) of the destination network and their year of connection is defined in the masterplan. The disconnected nodal demand is shifted to the newly connected parts, and the distribution is assumed to coincide to the final WDN, while the total demand is scenario dependent (see Section 2.1.4). By the fact that in a first step the intermediate WDN state are not individually designed, a “single state design” is followed. With this strategy critical WDN states are identified in space and time. In a second step a WDN re-design based on the least- “monetary” cost path is considered in case a specified performance cannot be assured (see Section 2.1.6). 

#### 2.1.4. Scenario Analysis

Future water demands and their geospatial distributions are highly uncertain [33]. They depend on multiple factors, including changes in population and consumption behavior, alterations in land use, and tourism [34]. Water loss reduction is a main challenge in WDN operation and management, saving valuable resources and providing additional flow capacities [35]. Furthermore, the water demand fraction, which is supplied via the central distribution system, depends on the degree of decentral supply (e.g., rainwater harvesting) or the provision of sufficient fire flow [36]. In this work we investigate possible future developments of the city’s total water demand by a scenario analysis, where the base demand is multiplied by time dependent factors [37]. 

#### 2.1.5. Simulation and Performance Assessment

The presented model has an interface to the hydraulic solver EPANET 2 [38] by which every network state is simulated for peak demand and a period of low water usage. Different daily patterns for domestic and industrial demands as well as for water losses are applied [39]. With the simulation result, global performance indicators are calculated to assess the hydraulic condition, the water quality and the reliability of the WDN. The following performance indicators are calculated: Nodal pressure head at peak demand, maximum water age during a low demand period, flow entropy at hourly peak demand, and the number of elementary loops of the created WDN layouts. 

The principles of global performance indicators (PIs) are briefly outlined. We use normalized performance indices, where each node *i* of the WDN is associated with a performance value from 0 (requirements not fulfilled) to 1 (requirements fulfilled), based on predefined threshold limits [40]. Equation (1) shows the calculation of the local nodal performance *PI_i_* with the upper and lower threshold values *T_u_* and *T_l_*. The factor α (0 or 1) depends on the performance criterion (minimum pressure or maximum water age), and *x_i_* is the performance variable (e.g., nodal pressure). In addition, a weight *w_i_* (e.g., nodal demand) is assigned to the local performances *PI_i_* and aggregated to one global representative value *PI*, where *N* is the total number of nodes (see Equation (2)).

(1) $$PI_{i}=\{\begin{array}{cc} \left|0-\alpha \right|, & x_{i}\leq T_{l}\\ \left|1-\alpha \right|, & x_{i}\geq T_{u}\\ \left|\frac{x_{i}-T_{l}}{T_{u}-T_{l}}-\alpha \right|, & x_{i}>T_{l}\wedge x_{i}<T_{u} \end{array}$$

(2) $$PI=\frac{\sum_{i=0}^{N}w_{i}\times PI_{i}}{\sum_{i=0}^{N}w_{i}}$$

For the WDN reliability assessment the informational flow entropy *E* is calculated. The concept is based on Shannon’s entropy function

(3) $$S=-\overset{nn}{\underset{l=1}{\sum }}p_{l}\ln p_{l}$$

where S is the entropy; $p_{l}$ is the probability of the *l*-th outcome; and *nn* is the number of outcomes [10]. S is zero, if one event is certain and the probabilities of the other events are zero. Tanyimboh and Templeman [41] developed a method to account for entropy in WDNs (Equation (4)).

(4) $$E=\underset{\begin{array}{c} E-supply\\ source\left(s\right) \end{array}}{\underbrace{-\overset{s}{\underset{k=1}{\sum }}\;\frac{Q_{0k}}{T}ln\left(\frac{Q_{0k}}{T}\right)}}-\underset{E-node\left(s\right)}{\underbrace{\overset{N}{\underset{i=1}{\sum }}\;\left(\frac{d_{i}}{T}ln\left(\frac{d_{i}}{T_{i}}\right)+\underset{ij\in ND_{i}}{\sum }\frac{Q_{ij}}{T}ln\left(\frac{Q_{ij}}{T_{i}}\right)\right)}}$$

In WDN analysis, *E* describes the uncertainty of all possible flow paths from the source to every demand node [11,42]. Usually, the number of sources is limited to one or a few supply nodes, confronting many demand nodes and, therefore, inhomogeneity in the flow distribution that exists. Also, the node degree (i.e., connected pipes to a junction) is limited through space [43], which influences entropy maximization. High entropy values indicate that a demand node is connected by many equally important flow paths, which characterizes highly reliable and redundant systems that can cope best with failures, such as pipe breaks [44]. Hence, in terms of WDN design, a high entropy value is sought. The value increases when the system is supplied by more sources of equal importance and when the flow paths have similar flows (absence of a “main” path). *E* consists of two terms, one describes the entropy related to the supply sources, and the other is related to the nodes. *N* is the total number of nodes, *s* denotes the number of sources, *T* is the total demand (≙total inflow to the WDN), *d_i_* is the demand at node *i*, *T_i_* is the total inflow at node *i*, *Q_ij_* is the outflow at node *i* to each successor node *j* (*ij* ∈ *ND_i_*, the pipe set of successors at node *i*) and *Q*_0*k*_ is the inflow from supply source *k* [13]. When, e.g., considering only one supply source, the first term in Equation (4) is zero. 

Moreover, the number of elementary loops in the network is assessed by *Γ = E − N + 1*, where *E* is the number of edges (pipes, valves, pumps) and *N* the number of nodes (junctions, tanks, reservoirs) of the WDN [43]. The various WDN design alternatives are assessed with a simple cost function which depends on the length and diameter of the pipes [13]. This allows for a cost-reliability comparison of different transition strategies, to support decision makers in choosing a reliable layout and design for the new WDN. Any other hydraulic modeling [13], network graph [32], or energy [45] based performance indicators can easily be implemented in the model.

#### 2.1.6. Critical Performance and Adaptation

**Figure 5:**
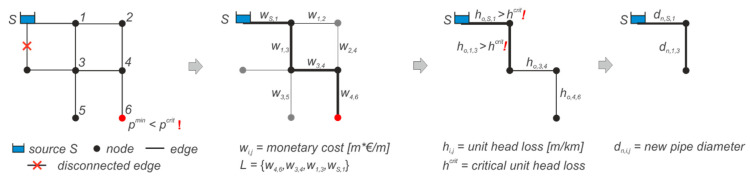
Local adaptation concept based on the redesign of critical pipes along the least- “monetary” cost path.

Existing WDNs can tolerate a certain amount of deviations from the optimum planning state to deal with uncertainties (e.g., demand increase, connectivity loss), whilst still guaranteeing sufficient performance requirements (e.g., sufficient pressure). In case the WDN cannot satisfy all requirements local adaptation measures (e.g., pipe replacement) must be considered to improve the performance to a sufficient level. For cost benefits, an effort is made to leave existing components of the WDN unchanged as much as possible. Therefore, we focus on the redesign and replacements of the weakest components by a diameter increase and roughness decrease, in case the minimum pressure threshold value *p^crit^* is not met. To find the weak network components, pipes with unusual high friction loss (user-defined threshold *h^crit^*) are identified and redesigned on a least- “monetary” cost path from the critical node to the supply source. The concept is shown in Figure 5 and subsequently described. The WDN can be mathematically represented as weighted graph *G*(*V*, *E(W*)), which consists of a node set *V*, an edge set *E* and a set of values *W* mapped. Dijkstra’s algorithm [46] is used to identify the shortest path *L* in the network from a critical node (e.g., minimum pressure *p^min^*) to a given root node (e.g., water source *S*). 

All edges *E* of the graph *G* are thereby weighted with the monetary costs *w_ij_(e_ij_),* where *e_ij_* is the edge between node *i* and *j* and *w_ij_* > 0. Along the shortest path, the pipes with unusual high unit head loss (*h_o,i,j_* > *h^crit^*) are identified and successively redesigned in an iterative process based on Equation (5), where new and old pipe diameters are denoted with *d_n,i,j_* and *d_o,i,j_*, *h_n_* and *h_o,i,j_* are the new and old unit head loss, respectively [47]. The following algorithm was implemented in MATLAB R2016b^®^.

(5) $$d_{n,i,j}=\sqrt[{5}]{\frac{d_{o,i,j}^{5}h_{o,i,j}}{h_{n}}}$$

The procedure is described as follows:

1. Start with the *n × n* adjacency matrix *A_ij_* from the finite graph WDN: *n* nodes; *e_ij_* edge from node *i* to node *j*; *E* all edges.
2. Weight each edge *e_ij_* with its diameter dependent monetary value *w_ij_*: *pipe length* [m] *× unit cost value* [€/m].
3. Identify the junction *J* with minimum pressure head *p^min^,* violating *p^crit^.*
4. Calculate the weighted shortest path *L* between source *S* and junction *J* using Dijkstra’s algorithm.
5. Determine unit head losses *h_ij_* of all edges in *E.*
6. Choose pipes from the shortest path *C* ⊆ *L*, which overstep the head loss threshold *h^crit^.*
7. If the no pipe oversteps *h^crit^*, reduce *h^crit^* by 10% and go back to step 6.
8. Design new diameter *d_n,i,j_* for each element in *C* with new unit head loss *h_n_* according Equation (5).
9. Round *d_n,i.j_* to nearest discrete diameter class and set pipe roughness to standard value for new pipes.
10. Replace all redesigned pipes in the WDN.
11. Run hydraulic simulation.
12. Update weights *w_ij_* in the adjacency matrix *A.*
13. Repeat step 3 to 10 until sufficient performance *p^min^ ≥ p^crit^* is reached in all nodes or the predefined number of maximum iterations is exceeded.
14. Determine (adaptation) costs.

This WDN redesign concept using the least cost path algorithm allows for a simple cost-effective pipe replacement of the weakest components of the WDN. This measure improves the pressure performances, but raises additional costs. All redesign and pipe replacement works are thereafter referred to as network adaptations. 

### 2.2. Case Study

Reliable demonstration cases for city transition are rare. We apply the presented methodology to a real world WDN transition, where a huge urban transition occurs within the next decades. The case study is the Swedish city of Kiruna, where substantial parts of the town have to be displaced about 4 km eastwards. The reason for that is the expansion of mining activities below ground, which threatens all infrastructure by creating underground settlements and cracks. Nevertheless, mining activities will continue. This rapid ongoing city transition is captured in the masterplan and is therefore well-suited to present the developed method. Although the above-ground infrastructure transition is precisely defined, the underground infrastructure is largely unknown. In Zischg et al. [48] the transition modeling of the urban drainage system was presented. 

#### 2.2.1. City Masterplan

**Figure 6:**
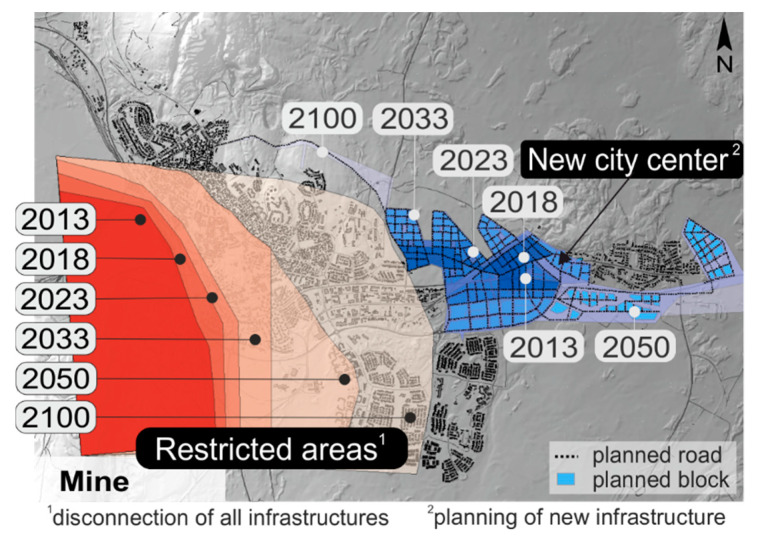
City masterplan of Kiruna defining the spatiotemporal urban transition: Restricted areas and newly planned city center with the future road network.

For the city transition of Kiruna, a detailed masterplan for future city development was provided by decision makers. It includes the planned road network and the building blocks of the new center, which is located at the eastern part of the current town. The construction and deconstruction processes are defined by six phases. People living in defined restricted areas have to be relocated to the new city center on a step-by-step basis by the end of the century (see Figure 6). 

#### 2.2.2. Source and Destination Networks

**Figure 7:**
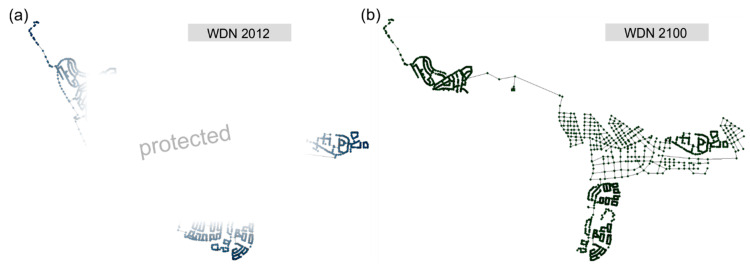
WDN states: (**a**) source network, and (**b**) one possible destination network-design alternative 1 (see Table 1).

The existing WDN of the year 2012 represents the source network and contains all necessary modeling parameters (see Figure 7a). For security reasons, the detailed layout cannot be shown here; nevertheless the quantitative results are presented. The WDN is set as the EPANET 2 input file and comprises 5845 junctions, 6069 pipes and three demand patterns for domestic and industrial consumption, as well as water losses. Water losses in the existing WDN are crucial, accounting for approximately 18% of the hourly peak demand (*Q_h,max_*). Despite their unknown distribution, they are integrated as additional nodal demand with the weighting of pipe length (linear) and nodal pressure (non-linear) according to Maskit and Ostfeld [49]. 

Figure 7b shows one possible destination network at year 2100. The new network parts are created based on the road network. The new systems are independently designed with a pipe sizing algorithm [31] and different future design demands ($f\times Q_{h,max}$). Comparing existing and future systems, the latter becomes denser (total networks length reduction of about 25%) and a new position of the water storage tank has to be considered. Due to the “unintended” disconnection of the north-western part of the network at year 2100, a new connection to the source has to be established. Based on the expansion target of the system a future safety factor *f* has to be chosen. It provides additional pipe capacity to deal with possible future demand increases and structural changes and to cope with other unexpected developments. The distribution of the demand nodes is identical for every design alternative (DA). In Table 1 the different design alternatives of the future WDN are defined and itemized from 1 to 20. The variation of the CI defines the degree of loops (alternative flow paths) of the new WDN parts. 

#### 2.2.3. Scenario Analysis

**Figure 8:**
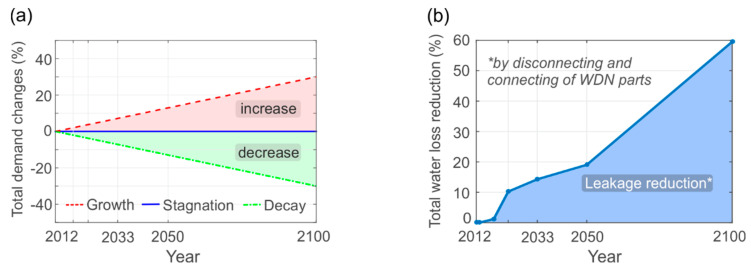
Water demand scenarios over time: (**a**) Future water consumption, and (**b**) water loss reduction.

The network transition analysis includes the investigation of three simple scenarios describing the total future water demand development, excluding water losses (see Figure 8a).

- Stagnation (Scenario 1): The basic assumption for the first scenario is that the total demand remains constant within the period of investigation. This describes no population change over time, but a change of its location.
- Growth (Scenario 2): The second scenario implies an increase of water usage up to 30% until the end of the century, which represents a population or demand per capita growth within the new city center [50].
- Decay (Scenario 3): The third scenario considers a gradual decrease of water usage up to 30% until the end of the century. This represents, a population decline, a demand per capita reduction, or an increased decentral water supply. For this case study, this scenario could also describe the effects of an economic collapse related to a falling market price of iron ore [50].

Along with the future water consumption, the water loss is another important factor to be considered. In the existing system the total water loss accounts for 42 l/s ($≙0.18\times Q_{h,max}$), which are to be significantly decreased in the future. Until 2100 the total water loss is expected to be reduced by 60% due to a newly constructed WDN of better quality (approximately 50–60% of total future network length) and the concurrent deconstruction of old, leaking system parts (see Figure 8b). This water loss reduction of approximately 25 l/s corresponds to the mean domestic demand equivalent of about 10,000 people (half the population in 2010), which shows the importance of considering water leakage in the existing system and reducing it in the future. A leakage increase of existing WDN parts over time and leakage reductions due to yearly pipe rehabilitations, are beyond the scope of our model. 

#### 2.2.4. Performance Assessment and Adaptation

The minimum pressure and entropy are investigated at maximum hourly water consumption, while water age is assessed for low demand periods. The user-defined threshold values *T_u_* and *T_l_* for the performance assessments of minimum pressure and water age are listed in Table 2. The critical performance is defined by the minimum pressure head of 15 m at a demand node at the maximum hourly peak load. In case this threshold is not fulfilled, the adaptation concept is applied. In this process, the critical pipes (see Section 2.1.6) with an exceeding unit head loss of 25 m/km (*h^crit^*) are redesigned using a new target unit head loss (*h_n_*) of 8 m/km. 

The diameter classes and unit costs used for the WDN design and adaptation for this case study application are adjusted from Ref. [13] and reported in Table 3.

## 3. Results

### 3.1. Model Application

In the following sections the previously introduced model is applied to the urban transition of Kiruna. Altogether, for the case study application 1440 WDN configurations are created and simulated, containing 20 generated design alternatives (different CIs and safety factors), each evaluated for six temporal states (from 2013 to 2100), under two hydraulic load conditions (high and low daily demand), three future demand scenarios, with and without adaptation. After the performance analysis and the application of the adaptation concept, a comparison of the 20 planning options is made by analyzing the costs and reliability indicators. 

#### 3.1.1. Possible Destination Networks

**Figure 9:**
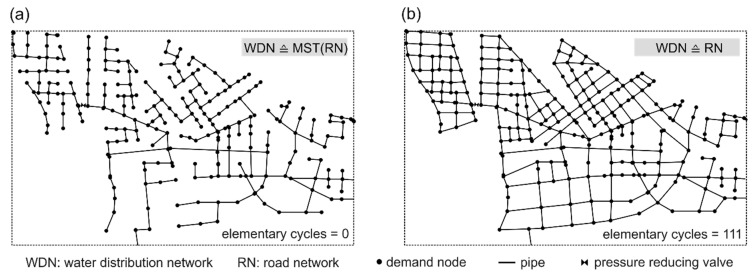
Example design alternatives considering different future WDN structures: (**a**) branched layout, and (**b**) strongly looped layout.

Figure 9 presents two example future WDN layouts. In Figure 9a a branched realization of the WDN to connect all demand nodes (CI = 0) is shown, which represents the minimum spanning tree (MST) of the co-located road network (WDN ≙ MST(RN)). Conversely, Figure 9b presents a highly looped WDN layout, which is identical to the co-located road network and, thus, has more alternative flow possibilities to connect the demand nodes (WDN ≙ RN). The nodal demand is taken from the expected population density map defined in the master plan. Water losses of newly constructed WDN parts are accounted with 10% of the average demand (referred to scenario 1). The position and setting of the pressure reducing valve (PRV) are determined manually on the basis of the topological characteristics of the road network and the topographic boundary conditions to ensure adequate pressure ranges of approximately 50 m in the new city center. The attributes of the PRV are unaltered for every design alternative. The new WDN parts are designed with a pipe sizing algorithm (see Section 2.1.2) and thus have sufficient performances. However, this does not necessarily mean that high performances are established across the entire system, due to integrated older WDN parts and different future demand scenarios. These interactions and possible weak points are investigated in the performance analysis, where intermediate WDN states are also considered.

#### 3.1.2. Intermediate State Networks

**Figure 10:**
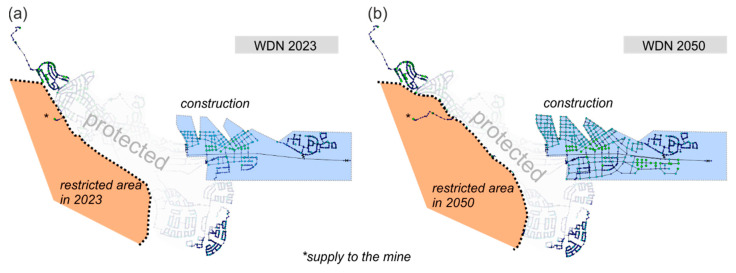
Example of two generated transition state WDNs for the Kiruna case study: (**a**) WDN 2023, and (**b**) WDN 2050.

The intermediate WDN states are defined by the years 2013, 2018, 2023, 2033 and 2050. During the transition process some parts of the existing WDN (source network) are disconnected, while new parts are connected at the same time. Simultaneously, a demand shift takes place from the disconnected parts to the new parts. “Unintended” network disconnections caused by the “intended” disconnections (i.e., pipes inside the restricted area) are of minor importance and therefore additionally removed. The demand of those parts is also shifted to the new WDN. Figure 10 shows two example intermediate state networks. Existing WDN parts cannot be fully shown for data protection and security reasons. 

#### 3.1.3. Performance Assessment, Critical Performance and Local Adaptation

Here we present the results of the performance assessment, where the WDN efficiencies are determined with global indices (see Section 2.1.5) during the transition process. In the first analysis no adaptation concept is considered, to identify transition states from which supply problems are to be expected, through the step-by-step stressing of the WDN. In the second analysis the adaptation concept, including the critical performance assessment and pipe redesign, is applied to provide sufficient performances and determine the necessary pipe replacement rates (see Section 2.1.6).

**Figure 11:**
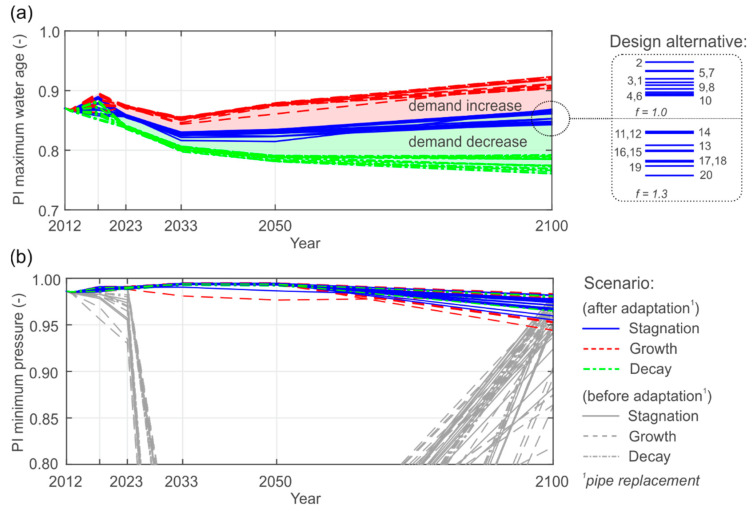
Investigation of performance trends considering: (**a**) The maximum water age during a low demand period, and (**b**) minimum pressure heads at hourly peak demand.

Figure 11 illustrates the performance trends of the maximum water age and minimum pressure from the initial state in 2012 to the final state in 2100. The PI minimum pressure for the source network is 0.986. This means that the pressure head at approximately 99% of the demand nodes is greater or equal to 40 m for hourly peak demand (*Q_h,max_*). 

In particular Figure 11a shows the trend of water age performance, where slight drops at the intermediate states are observed, even for the stagnation scenario of constant total demand. The reason for that are excess capacities at intermediate states, due to faster connection than disconnection process (shifted demand from the restricted areas is lower than the design capacities of the newly connected pipes). We also observe a strong dependency on the future water consumption, where the water age is lower (desired) for a demand increase. The effect of the design alternative of the new center on the water age reveals less influence. We do not see a correlation between the CI variation and the water age, however using higher safety margins (*f* = 1.3) for increased water consumption reveals lower water age performances due to lower flow velocities. 

**Figure 12:**
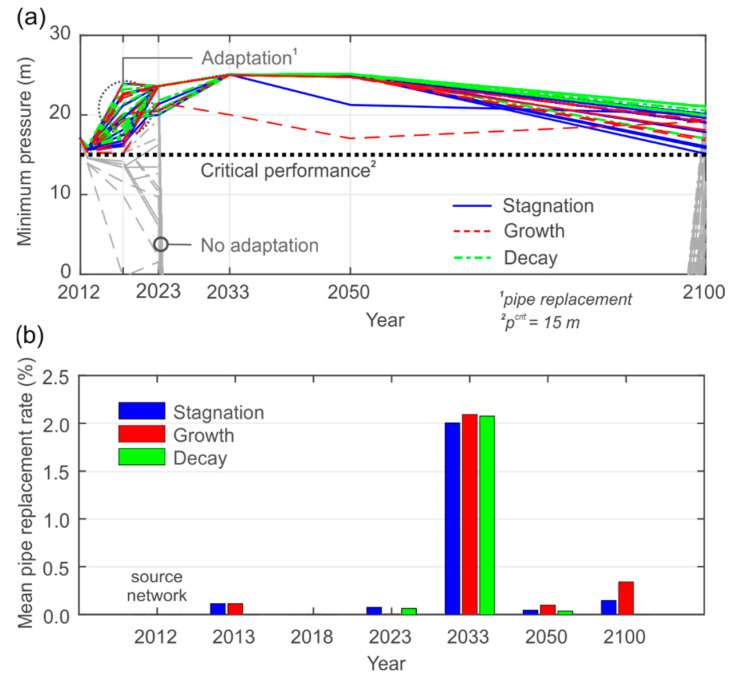
Adaptation concept: (**a**) Performance analysis based on minimum pressure head criterion, and (**b**) the necessary pipe replacement to achieve a sufficient performance level.

Figure 11b presents the minimum pressure performances with (highlighted) and without (shaded) application of the adaptation concept. It can be seen that until state 2023 the WDN is relatively robust when facing connectivity losses and changed flow patterns. However, after state 2023, the first weak points occur where the system breaks down due to overused pipe capacities, which are mainly caused by the disconnection of pipes. This is noted by the relatively similar performance drops, regardless of the demand scenario. With application of the adaptation concept and the redesign of the weakest pipes, the minimum pressure performances significantly improve to guarantee sufficient hydraulic conditions at peak demand. At final state, higher variations of the PI minimum pressure can be observed, caused by greater demand variations, different WDN topologies and pipe replacement rates (see Figure 12). Owing to the fact the main problems occur in the existing WDN parts, there are also no clear distinctions here between the design alternatives that can be made. 

We then take a closer look on the results of the critical performance analysis and the local system adaptation (according to Section 2.1.6). Figure 12a exemplarily presents the local minimum pressure head at the most unfavorable demand node before (shaded) and after system adaptation (highlighted), whereas in Figure 11 global indices were shown. The threshold value *p^crit^* for the critical performance analysis is shown by the bold dotted line in Figure 11. The adaptation concept is performed when *p^crit^* is violated at hourly peak demand. The minimum pressure head in the source network is approximately 17 m. The performance pathways are similar to the global minimum pressure indicator, with the highest variation at the final state. Corresponding pipe replacement rates are demonstrated in Figure 12b, differentiated by the demand scenario. For the growth scenario the highest replacement rates (2.7%) are necessary, whereas they are lowest for the decay scenario (2.2%). However, pipe replacement is necessary for all tested pathways and the differences between the scenarios are relatively low, which indicates that the pipe disconnections represent the main cause of stress for the WDN, especially after the year 2023. One reason for the relatively low adaptation rates is the step-by-step reduction of water losses (see Figure 8b), which provides additional capacities for domestic and industrial water demand. Performed pipe replacements at state *t*, influence the next future state *t+1.* This explains, for example, why for the growth scenario in 2023 no pipe upgrades are performed, as they have already been completed at an earlier state. By using this adaptation concept, the latest possible times for pipe replacement are chosen to maintain sufficient performances. The accumulated adaptation rates between 2–3% (corresponds on average to 0.03% per year) of the total network length are low compared to the necessary pipe rehabilitation of approximately 1–2% per year [51]. The obtained information about the locations of the weakest WDN parts (not shown here for security reasons), the amount, and the latest possible time for necessary pipe replacements, can support decision makers in planning necessary rehabilitation works.

#### 3.1.4. Cost-Reliability Comparison of the Design Alternatives

**Figure 13:**
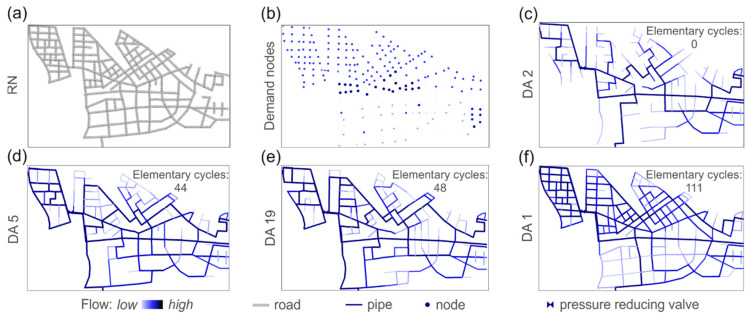
Chosen design alternatives (DAs) and flow patterns at final state 2100 for Kiruna’s new city center. (**a**) Road network, (**b**) demand distribution, and (**c**–**f**) four design alternatives (DAs) with varying network topology and flow distribution.

The last planning objective is to identify a reliable and cost-efficient future WDN layout. From the results presented in the previous section, no conclusions about the reliability of the different design alternatives could be made. The design alternatives differ in total network length, pipe diameters and required adaptation works, which all can be associated with costs. For WDN reliability and robustness assessment, the elementary number of loops and the flow entropies are calculated. Figure 13 shows example WDN alternatives for Kiruna’s new city center. Road network (Figure 13a), demand distribution (Figure 13b), and four design alternatives (DAs) with varying network topology and flow distribution are illustrated. Darker links indicate high flows, while brighter links have lower flows. 

**Figure 14:**
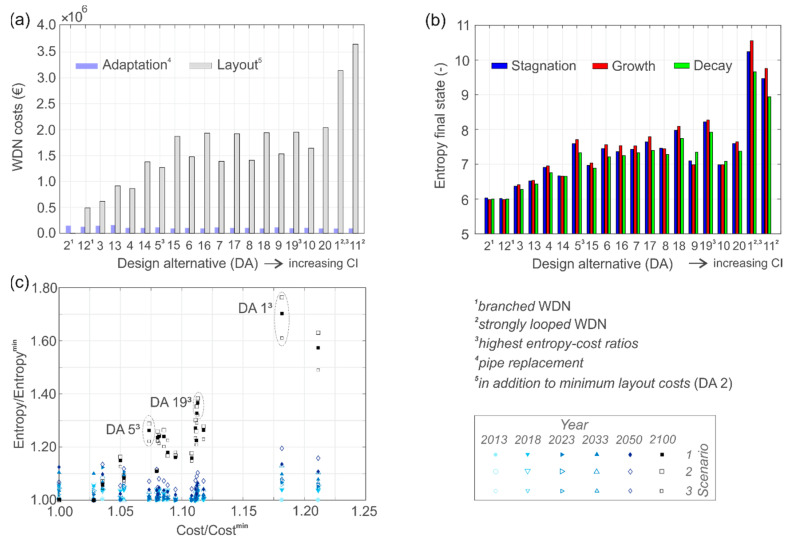
Comparison between (**a**) WDN costs, and (**b**) flow entropy at final state; (**c**) shows the temporal scale of the entropy-cost relation for 6 WDN states.

Figure 14a compares the associated costs for the newly built WDN (referred as “layout costs”) and the adaptation costs (associated with the necessary pipe replacement for sufficient hydraulic performance) for the 20 design alternatives. Their loop degree (measured with the CI) increases from left to right on the abscissa. The “layout costs” are defined as the additional costs compared to the cheapest layout option, which represents the branched WDN with zero elementary cycles (see Figure 13c) and no additional safety capacities (DA 2). The strongly looped WDN, which is identical to the RN layout with an additional safety margin of 30% for future demand increase, is the most expensive planning option (DA 11). The reason for that is the highest total network length and high flow capacities. Furthermore, it can be observed that the adaptation costs are slightly higher for lower loop degree, but it is remarkable that the relative layout costs are dominating the adaptation costs for this case study. 

Figure 14b illustrates the calculated flow entropy values for the 20 design alternatives under three future demand scenarios at the final WDN state. It can be seen that branched networks with no alternative flow paths (CI = 0) are the most cost-effective options, but reveal the lowest entropy values. When the WDN layout is identical to the RN, the entropies are highest, but also the costs strongly increase. Moreover, it can be noted that bigger pipe diameters (due to higher safety factors), generally cannot be associated with higher entropies. Take, for example, the diameter increase of a main pipe. This would consequently attract more flow, cause higher flow inhomogeneity, and thus reduce the entropy. Furthermore, it can be observed that, the higher the loop degree, the higher is the entropy variation for the different demand scenarios. The reason for this increased variability is flow rearrangements, which alter the flow distributions. For most design alternatives the growth scenario reveals the highest entropies, except for the design alternatives 2, 12, 14, 8, 9 and 10. 

When comparing the (normalized) entropy-cost relation in Figure 14c, a strong correlation can be observed for the final state WDNs, for which the systems are designed for. At earlier states no entropy-cost relation is found, due to the dominance of existing WDN parts (“unchanged topology”) and only minor newly constructed parts (“changing topology”). Highest entropy-cost ratios at final state, and thus the most suitable WDN options for Kiruna’s new city center, are the design alternatives 5, 19 and 1, with 44, 48 and 111 elementary loops, respectively (see Figure 13d–f). 

## 4. Discussion

In the following we discuss the implications and limitations of the presented work, and address possible future directions.

- Flow entropy as a reliability measure: For every flow configuration one specific entropy value exist and therefore depends on the source and demand node distribution and the network topology. Most studies on entropy maximization considered the redistribution of demand loads, whereas the topology and flow direction were assumed to be fixed. When considering the temporal scales of WDN (e.g., transitioning) this is not probable. We considered variations in the network topology and total demand of a real case study and found correlations between the loop degree and entropy. We think that further studies of spatial and topological network characteristics are necessary (e.g., the existence of loops in high capacity pipes or the spatial distribution of demand nodes with the source nodes) to better understand the entropy scaling of complex real-world systems.
- “In-silico” networks: Unknown WDN parts are approximated with surrogate network data (e.g., roads) and complement known/existing data. This method has great potential in data reconstruction, network disaggregation, and network growth processes, such as network densification and/or expansion. Furthermore, certain links of the co-located network might be excluded in the generation process, so that critical components do not overlay in space (e.g., main pipe underneath main road). Those topics will be part of further investigations.
- WDN design: After the WDN layout was set, the fast pipe design algorithm proposed by [31] was used. However, any other WDN optimization algorithms could be integrated if feasible in terms of computational costs. The distribution of demand nodes was fixed for every generated WDN layout, only their weights were changed for the WDN design and the scenario assessment.
- Urban transition: We demonstrated the method for a very drastic city transition, where approximately half of the network will change in the future. However, the method works also for slower transitions (including network densification or network shrinking). Here, the urban development and therefore the network change is defined in a masterplan. In cases where no masterplan is available, stochastic models to predict the network development (e.g., preferential or other fitness-based attachment models) can be applied. In future work different case study applications could be tested.
- Performance assessment: We mainly focused on the minimum pressure and the maximum water age as performance indices. The reliability was assessed with the flow entropy. The maximum pressure, flow velocities and modified resilience [13] were also considered, but not shown in this paper because no additional insight was gained. Additional topological, energy or hydraulic indices could easily be integrated without changing the proposed method. From a sustainability perspective, advanced pressure management, e.g., to reduce pipe leakage [52] and the use of pump as turbines instead of PRVs [53], could also be of great interest for WDN transitioning. However, these investigations were not subject of this work.
- Local adaptation concept: When the critical performance (i.e., minimum pressure) cannot be fulfilled during the transition, specific pipes are redesigned and replaced. Pipe replacement is only one of many possible adaptation measures to provide sufficient performances. Often described as a “hard” measure, it raises additional costs. From the authors point of view this approach is especially useful when the necessary pipe replacement can (partly) coincide with yearly necessary rehabilitation works, i.e., the pipe must be replaced anyway. When the age of the pipes is known, the consideration of installing parallel pipes could be a suitable alternative option to increase capacity. Alternatively, “soft” measures, like peak demand reduction or decentral solutions of rainwater harvesting, might be more beneficial from a sustainability point of view.
- Least cost path for pipe redesign: In branched networks there is one defined path from a demand node (e.g., critical node with the worst performance) to the water source. In looped networks more than one path exists. By calculating the least- “monetary” cost path, short connections with small diameter are candidate pipes for the redesign. Depending on data availability of the source network, the least cost paths could be calculated by using alternative edge weights (e.g., pipe age, roughness or material). The presented algorithm requires a sufficient geodetic head difference between water source and demand node, since only the head losses are reduced and no pumps are considered.

## 5. Summary and Conclusions

In this work, a novel model was presented to assess complex urban transitions and their effects on the water distribution system under a variety of uncertainties. In contrast to the work presented in Zischg et al. [22], here the interaction of existing WDN and approximated “in-silico” networks are addressed over time while also taking into account uncertain population developments. Informational flow entropy and other metrics are calculated on the pathway from an initial to a possible future WDN state to assess the temporal variability and the suitability of future planning options. Accompanying effects of external stresses, such as simultaneous connectivity loss, network expansion, and demand changes were considered. In the following the main points of the research are summarized:

- Unknown future WDN parts were generated based on the co-located known road network. In this process several network layouts (ranging from the minimum spanning tree to strongly looped networks) were created and connected with the existing WDN. These WDN parts were designed with a simple and fast pipe-sizing algorithm using a defined pressure surface inclination at hourly peak demand.
- A city masterplan that defines the urban transition was used to change the WDN. Step-by-step WDN parts were disconnected, while other parts were connected to the existing (source) network. The functional changes assessed with hydraulic, water quality and reliability performance indices and reveal critical WDN configurations where problems are to be expected. For the case study application, it was shown that after the year 2023 pressure deficits will occur when appropriate transition planning is neglected.
- A local adaptation concept was successfully applied in which critical pipes on the least- “monetary” cost path was redesigned, if the required minimum pressure head could no longer be guaranteed by the stressed system. With this targeted pipe replacement, the minimum pressure requirements were established across all transition states with overall replacement rates of approximately 3%.
- Best suited WDN design alternatives were identified with a temporal cost-reliability (measured with the flow entropy) comparison. Generally, higher flow entropy values were achieved for costly planning options, where additional and equally important flow paths were provided especially for high capacity pipes. Highest entropy-cost ratios were obtained for network layouts with different loop degree.

The implications of the results are performance trends for various possible WDN planning options during a transition process from an initial to a desired future state. As a result, decisionmakers can be supported at an early phase of the project to plan necessary rehabilitation works by knowing the hydraulic deficiencies in space and time, in order to construct a cost-efficient and reliable future water supply system. 

## Figures and Tables

**Table 1:** Definition of design alternatives considering different cycle indices and safety factors.

Safety Factor *f*	Cycle Index CI (-)									
(-)	max *	0 **	0.1	0.2	0.3	0.4	0.5	0.6	0.7	0.8
1	1	2	3	4	5	6	7	8	9	10
1.3	11	12	13	14	15	16	17	18	19	20

* WDN layout is identical to the road network (RN); ** WDN layout represents the minimum spanning tree (MST) of the RN.

**Table 2:** User-defined threshold values for performance analysis.

Performance Threshold	*T_u_*	*T_l_*	α
Minimum pressure (m)	40	0	0
Maximum water age (h)	120	24	1

**Table 3:** Pipe diameters *D* and unit costs, adopted for the design phase.

*D* (mm)	Costs (€/m)	*D* (mm)	Costs (€/m)	*D* (mm)	Costs (€/m)
50	190	125	250	300	360
80	227	150	272	350	399
90	229	160	275	400	420
100	231	200	299	450	450
110	235	250	328	500	480

## References

[1] Oswalt P., Rieniets T. (2006). Altas of Shrinking Cities.

[2] Ryan B.D., Campo D. (2013). Autopia’s end: The decline and fall of Detroit’s automotive manufacturing landscape. J. Plan. Hist..

[3] The Telegraph The World’s 20 Fastest Growing Cities. https://www.telegraph.co.uk/travel/lists/fastest-growing-cities-in-the-world/fastest1/.

[4] Sitzenfrei R., Möderl M., Rauch W. (2013). Assessing the impact of transitions from centralised to decentralised water solutions on existing infrastructures—Integrated city-scale analysis with vibe. Water Res..

[5] Tscheikner-Gratl F., Sitzenfrei R., Rauch W., Kleidorfer M. (2015). Enhancement of limited water supply network data for deterioration modelling and determination of rehabilitation rate. Struct. Infrastruct. Eng..

[6] Sempewo J.I. (2012). Transitioning of Urban Water Distribution Systems.

[7] Khatri K., Vairavamoorthy K. A new approach of risk analysis for complex infrastructure systems under future uncertainties: A case of urban water systems. Proceedings of the Vulnerability, Uncertainty, and Risk.

[8] Brown R., Keath N., Wong T. Transitioning to water sensitive cities: Historical, current and future transition states. Proceedings of the 11th International Conference on Urban Drainage.

[9] Sitzenfrei R., Möderl M., Mair M., Rauch W. (2012). Modeling dynamic expansion of water distribution systems for new urban developments. Proceedings of the World Environmental and Water Resources Congress 2012.

[10] Shannon C.E. (1948). A mathematical theory of communication. Bell Syst. Tech. J..

[11] Tanyimboh T.T. (2017). Informational entropy: A failure tolerance and reliability surrogate for water distribution networks. Water Resour. Manag..

[12] Gheisi A., Naser G. (2015). Multistate reliability of water-distribution systems: Comparison of surrogate measures. J. Water Resour. Plan. Manag..

[13] Creaco E., Fortunato A., Franchini M., Mazzola M.R. (2013). Comparison between entropy and resilience as indirect measures of reliability in the framework of water distribution network design. Procedia Eng..

[14] Marlow D.R., Moglia M., Cook S., Beale D.J. (2013). Towards sustainable urban water management: A critical reassessment. Water Res..

[15] Eggimann S., Truffer B., Maurer M. (2015). To connect or not to connect? Modelling the optimal degree of centralisation for wastewater infrastructures. Water Res..

[16] Urich C., Rauch W. (2014). Exploring critical pathways for urban water management to identify robust strategies under deep uncertainties. Water Res..

[17] Creaco E., Franchini M., Walski T. (2013). Accounting for phasing of construction within the design of water distribution networks. J. Water Resour. Plan. Manag..

[18] Creaco E., Franchini M., Walski T. (2015). Taking account of uncertainty in demand growth when phasing the construction of a water distribution network. J. Water Resour. Plan. Manag..

[19] Van der Brugge R., Rotmans J. (2007). Towards transition management of european water resources. Water Resour. Manag..

[20] Sempewo J., Vairavamoorthy K., Grimshaw F. (2010). Transitioning of urban water distribution systems. Proceedings of the World Environmental and Water Resources Congress 2010.

[21] Mair M., Rauch W., Sitzenfrei R. (2014). Improving incomplete water distribution system data. Procedia Eng..

[22] Zischg J., Mair M., Rauch W., Sitzenfrei R. (2017). Enabling efficient and sustainable transitions of water distribution systems under network structure uncertainty. Water.

[23] Sitzenfrei R., Möderl M., Rauch W. (2013). Automatic generation of water distribution systems based on GIS data. Environ. Model. Softw..

[24] Gong J., Chen W., Liu Y., Wang J. (2014). The intensity change of urban development land: Implications for the city master plan of Guangzhou, China. Land Use Policy.

[25] Ferguson B.C., Frantzeskaki N., Brown R.R. (2013). A strategic program for transitioning to a water sensitive city. Landsc. Urban Plan..

[26] Kuzniecow Bacchin T., Ashley R., Sijmons D., Zevenbergen C., Van Timmeren A. Green-blue multifunctional infrastructure: An urban landscape system design new approach. Proceedings of the ICUD 2014 13th IAHR/IWA International Conference on Urban Drainage.

[27] Bell M., Perera S., Piraveenan M., Bliemer M., Latty T., Reid C. (2017). Network growth models: A behavioural basis for attachment proportional to fitness. Sci. Rep..

[28] Klinkhamer C., Krueger E., Zhan X., Blumensaat F., Ukkusuri S.V., Rao P.S.C. (2017). Functionally fractal urban networks: Geospatial co-location and homogeneity of infrastructure. arXiv.

[29] Mair M., Zischg J., Rauch W., Sitzenfrei R. (2017). Where to find water pipes and sewers?—On the correlation of infrastructure networks in the urban environment. Water.

[30] Sitzenfrei R., Mair M., Diao K., Rauch W. (2014). Assessing model structure uncertainties in water distribution models. Proceedings of the World Environmental and Water Resources Congress 2014.

[31] Saldarriaga J., Takahashi S., Hernández F., Escovar M. (2011). Predetermining pressure surfaces in water distribution system design. Proceedings of the World Environmental and Water Resources Congress 2011.

[32] Bollobás B. (2013). Modern Graph Theory.

[33] Kang D., Lansey K. (2014). Multiperiod planning of water supply infrastructure based on scenario analysis. J. Water Resour. Plan. Manag..

[34] Donkor E., Mazzuchi T., Soyer R., Alan Roberson J. (2014). Urban water demand forecasting: Review of methods and models. J. Water Resour. Plan. Manag..

[35] Puust R., Kapelan Z., Savic D.A., Koppel T. (2010). A review of methods for leakage management in pipe networks. Urban Water J..

[36] Sitzenfrei R., Zischg J., Sitzmann M., Bach M.P. (2017). Impact of hybrid water supply on the centralised water system. Water.

[37] Basupi I., Kapelan Z. (2013). Flexible water distribution system design under future demand uncertainty. J. Water Resour. Plan. Manag..

[38] Rossman L.A. (1999). The EPANET programmer’s toolkit for analysis of water distribution systems. WRPMD’99: Preparing for the 21st Century.

[39] Diao K., Barjenbruch M., Bracklow U. (2010). Study on the impacts of peaking factors on a water distribution system in germany. Water Sci. Technol..

[40] Möderl M., Lukas A., Hellbach C., Rauch W., Sitzenfrei R., Mayr E., Perfler R., Mair M. (2011). GIS based applications of sensitivity analysis for water distribution models. Proceedings of the World Environmental and Water Resources Congress 2011.

[41] Tanyimboh T.T., Templeman A.B. (1993). Optimum design of flexible water distribution networks. Civ. Eng. Syst..

[42] Setiadi Y., Tanyimboh T.T., Templeman A.B. (2005). Modelling errors, entropy and the hydraulic reliability of water distribution systems. Adv. Eng. Softw..

[43] Barthélemy M. (2011). Spatial networks. Phys. Rep..

[44] Di Nardo A., Greco R., Santonastaso G.F. Synthetic indices of robustness of water distribution networks. Proceedings of the 11th International Conference on Computing and Control for Water Industry (CCWI).

[45] Cabrera E., Pardo M.A., Cobacho R., Cabrera E. (2010). Energy audit of water networks. J. Water Resour. Plan. Manag..

[46] Deng Y., Chen Y., Zhang Y., Mahadevan S. (2012). Fuzzy dijkstra algorithm for shortest path problem under uncertain environment. Appl. Soft Comput..

[47] Gujer W. (2007). Urban Water Management.

[48] Zischg J., Goncalves M.L., Bacchin T.K., Leonhardt G., Viklander M., van Timmeren A., Rauch W., Sitzenfrei R. (2017). Info-gap robustness pathway method for transitioning of urban drainage systems under deep uncertainties. Water Sci. Technol..

[49] Maskit M., Ostfeld A. (2014). Leakage calibration of water distribution networks. Procedia Eng..

[50] Gunn A., Rogers B., Urich C. (2016). Identification and Assessment of Long-Term Green/Blue Drainage Strategies for Kiruna, Sweden.

[51] Scholten L., Scheidegger A., Reichert P., Mauer M., Lienert J. (2014). Strategic rehabilitation planning of piped water networks using multi-criteria decision analysis. Water Res..

[52] Stokes J.R., Horvath A., Sturm R. (2013). Water loss control using pressure management: Life-cycle energy and air emission effects. Environ. Sci. Technol..

[53] Carravetta A., Houreh S.D., Ramos H.M. (2017). Pumps as Turbines: Fundamentals and Applications.

